# Delirium in COVID-19: An Atypical Case Managed With Quetiapine

**DOI:** 10.7759/cureus.25776

**Published:** 2022-06-09

**Authors:** Mark Laurence Silva, Owais Gul, Vamsi Priya Aravally, Maria Khalid, Tad Williams

**Affiliations:** 1 Internal Medicine, United Health Services Wilson Medical Center, Johnson City, USA; 2 Internal Medicine, Shaheed Zulfiqar Ali Bhutto Medical University, Islamabad, PAK; 3 Pharmacy, United Health Services Wilson Medical Center, Johnson City, USA

**Keywords:** atypical antipsychotics, atypical presentation, quetiapine, covid-19, delirium

## Abstract

Coronavirus disease 2019 (COVID-19) can present without the typical symptoms of respiratory tract infection. Delirium has been reported as a prominent feature leading to an atypical presentation in older adults infected with COVID-19. Here, we present the case of a 65-year-old female who came to our hospital with confusion and altered mental status. The patient maintained an asymptomatic course of illness after testing positive for COVID-19 two weeks prior to the hospital visit. An appropriate workup was done to rule out other causes of the patient’s symptoms. During the next couple of days, the patient developed classic symptoms suggestive of delirium. The patient was eventually treated based on the general guidelines for delirium management due to the absence of adequate medical literature specifying the management of delirium in the population of interest. Thus, the patient was given a trial of an atypical antipsychotic, quetiapine, to which she responded well and was subsequently discharged one week later.

## Introduction

Coronavirus disease 2019 (COVID-19), caused by severe acute respiratory syndrome coronavirus 2 (SARS-CoV-2), ranges from mild, moderate, to severe clinical symptoms which typically include fever, cough, dyspnea, malaise, headache, anosmia, sore throat, congestion, nausea, vomiting, and/or diarrhea [1]. Emerging evidence has revealed delirium as an atypical clinical presentation of COVID-19 infection in the absence of upper respiratory symptoms [2-4]. According to a multicenter retrospective cohort study, up to 28% of older COVID-19-infected patients presented with delirium, with or without the typical symptoms of COVID-19, and were significantly associated with worse hospital outcomes including death [3]. Consequently, patients presenting with delirium should prompt clinicians for early testing to guide in the management of COVID-19. To date, there are limited evidence-based pharmacological recommendations to manage delirium in COVID-19 patients. Current guidelines employed for the management of delirium in COVID-19 were extrapolated from anecdotal experiences; either from the general delirium treatments or from the guidelines used during the severe acute respiratory syndrome (SARS) and Middle Eastern respiratory syndrome (MERS) epidemics [1]. We present a case of an asymptomatic patient infected with COVID-19 who subsequently developed delirium and was managed with an atypical antipsychotic, quetiapine.

## Case presentation

A 65-year-old female who used to work at a long-term care facility presented to the emergency department via emergency medical services (EMS) after her family members noticed an acute change in her mental status. She had tested positive for COVID-19 approximately two weeks before the presentation but had remained asymptomatic until she developed mental status changes. On her way to the hospital, the EMS team noticed repeated bouts of confusion accompanied by disorientation to time and place. Her medical history was significant for hypertension and lumbar spinal degeneration. Her home medications included hydrochlorothiazide 12.5 mg capsule once daily, metoprolol succinate 100 mg tablet once daily, and gabapentin 600 mg tablet twice daily.

On presentation, vital signs were normal. Initial physical and neurological examinations were unremarkable. The mental status assessment revealed that the patient was disoriented, unable to repeat or recall words, and failed to follow simple commands. Speech-wise, the patient sounded confused with no ifs or buts noted in her spoken sentences. Initial labs were notable for hypokalemia with a serum potassium level of 2.8 mEq/L (normal range: 3.5-5.5 mEq/L), which was adequately replaced. The rest of the metabolic panel and hemogram was normal. Her C-reactive protein was found to be 3.4 mg/dL (normal: <0.9 mg/dL), an elevation likely suggestive of the ongoing COVID-19 infection. Urinalysis, serum thyroid-stimulating hormone, and serum vitamin B12 levels were within normal limits. Blood and urine cultures did not show any evidence of infection. Chest X-ray revealed some bibasilar lung infiltrates that appeared unchanged from the previous picture obtained at the time of COVID-19 diagnosis. A non-contrast computed tomography scan of the brain was negative for any acute intracranial pathology. The patient was admitted to the hospital floor, and daily monitoring of her labs was initiated. Her gabapentin was stopped due to its known neuropsychiatric side effects, and hydrochlorothiazide was held temporarily for hypokalemia. However, in the subsequent days, she developed nocturnal restlessness which progressed to frank agitation and combativeness. The patient was diagnosed with classic delirium based on the Diagnostic and Statistical Manual of Mental Disorders, Fifth Edition criteria, and no possible etiology could be identified except the pre-existing COVID-19 pneumonia. She failed to respond to conservative management with a reorientation strategy and arrangement of bedside sitters. She was subsequently started on oral quetiapine 25 mg nightly and intravenous haloperidol 2 mg as needed every six hours. This did not result in any improvement and prompted a consult with the hospital’s psychiatry service. Her medication regimen was then revised to oral quetiapine 50 mg one tablet in the morning, one tablet in the afternoon, and a 100 mg tablet every night before sleep. In addition, she was provided with oral quetiapine 50 mg as needed every six hours for frank agitation. The patient’s symptoms gradually subsided and she was safely discharged home after one week.

## Discussion

Delirium is defined as a sudden change in mental function that is most often transient and reversible. It typically involves disturbances of attention, disorganized thinking, and an altered level of consciousness that fluctuates throughout the course of the day. Depending upon the subtype, delirium can be referred to as hypoactive if characterized by weakness and lethargy along with the presence of cardinal symptoms. However, it can also manifest as agitation, delusions, hallucinations, or combative restless behavior observed frequently in the hyperactive subtype. Hyperactive presentations may account for up to 25% of all delirium cases but some patients demonstrate a mixed subtype with overlapping clinical features. Delirium in COVID-19 patients can be attributed to numerous causes, including the presence of multiple comorbid conditions, injudicious medication use, old age, social isolation whether in a hospital or home quarantine, and virus-induced or immune-mediated damage to the nervous system [5,6].

The underlying pathophysiology leading to delirium in COVID-19 infection is multifactorial and not fully understood. It has been postulated that the inflammatory response to the infection disrupts the blood-brain barrier that helps the virus to reach the central nervous system, migrate to the neuronal pathways, and infect the nerve endings resulting in neuroinflammation [7,8]. Alterations in the neurotransmitter levels found to be associated with delirium include an excess of dopamine, as well as serotonin and decreased acetylcholine production [9]. In addition, social isolation due to reduced patient contact to mitigate the spread of infection can contribute to the worsening of delirium [10].

To date, no randomized controlled trial has evaluated the treatment of delirium specifically in COVID-19 patients. As for consensus, behavioral modifications are the first line in the management of delirium followed by pharmacological treatments if behavioral strategies are not sufficient to control the patient’s behavior or ensure the safety measures. Our case report focused on the pharmacologic management of an asymptomatic COVID-19-infected patient who later developed acute hyperactive delirium using an atypical antipsychotic, quetiapine. Quetiapine has been frequently used in intensive care units and inpatient medical units for the management of delirium in hospital settings [11]. It works by antagonizing serotonin 5-hydroxy-tryptamine (5-HT2A), dopaminergic D1 and D2, histamine H1 and H2, and adrenergic α1 and α2 receptors, as well as acting as a partial agonist at the serotonin 5-HT1A receptors [1,12]. We postulate that quetiapine aided in the treatment of COVID-19-induced delirium due to its effect on modulating dopaminergic and serotonergic actions. Stimulation of the 5-HT1A receptors has an inhibitory downstream effect on neuronal firing. Agonists of this receptor group are predicted to reduce anxiety, aggression, and impulsivity. In contrast, agonism of the 5-HT2A receptors is stimulatory in nature and increases the downstream firing. Antagonism of these receptors may complement the inhibitory effect of 5-HT1A receptor signaling. While traditional antipsychotics such as haloperidol are used chiefly for their anti-dopaminergic effect leading to the inhibition of central D2 receptors, the atypical antipsychotics are differentiated by their ability to also target the central serotonin receptors. Multiple serotonin receptor subtypes have been identified throughout the central nervous system (CNS), and some of their roles are better elucidated than others. It is postulated that the partial antagonism of D2 receptors along with the antagonism of 5-HT2A receptors explains the effectiveness of the atypical antipsychotics in treating schizophrenia and depression, as well as causing mood stabilization in bipolar disorder. The bipartite 5-HT1A-5-HT2A model also demonstrates that serotonin has a role to play along with dopamine in affecting the behavioral elements of psychosis and that modulation of the serotonin receptors can also complement the modulation of the dopamine receptors. There is a variation among the atypical antipsychotics in both the affinity for, and the number of, various neurotransmitter receptors impacted. Given the varying affinity for different receptor types, it should be noted that the expected effects of quetiapine will depend significantly on the dosage used. At lower doses, quetiapine is effectively an expensive antihistamine. It requires doses greater than 100 mg daily to be effective for depression, and even higher doses to be effective as an antipsychotic [12,13]. Our patient, therefore, received a total dosage of 400 mg as scheduled along with as-needed doses of quetiapine. Her agitation gradually subsided, and she was safely discharged home after one week of treatment. Quetiapine is recommended as one of the preferred medications over the typical antipsychotics such as haloperidol because it has a lower risk of developing extrapyramidal side effects [14,15]. However, quetiapine also demonstrates some anticholinergic side effects. Notably, quetiapine exhibits drug-drug interaction with CYP3A4 inhibitors including protease inhibitors used in human immunodeficiency virus, some of which were also previously used as proposed treatments for COVID-19 [15,16]. Nevertheless, more research is needed to determine the rationale for using atypical antipsychotics such as quetiapine for the pharmacological management of delirium in patients with COVID-19.

## Conclusions

Delirium can be an atypical presentation of COVID-19 infection. The pathophysiology leading to delirium among COVID-19 patients is not fully understood yet. However, excessive dopamine and serotonin levels can be countered by atypical antipsychotics such as quetiapine. However, with limited guidelines available to manage delirium specifically related to COVID-19 infections, a generalized approach is being used currently with more room available for further research and analysis.
